# Supplementary figures and images for: A human-neutral large carnivore? No patterns in the body mass of gray wolves across a gradient of anthropization
Source: PLoS One. 2023 Jun 1;18(6):e0282232. doi: 10.1371/journal.pone.0282232 (PMC10234544; doi:10.1371/journal.pone.0282232)

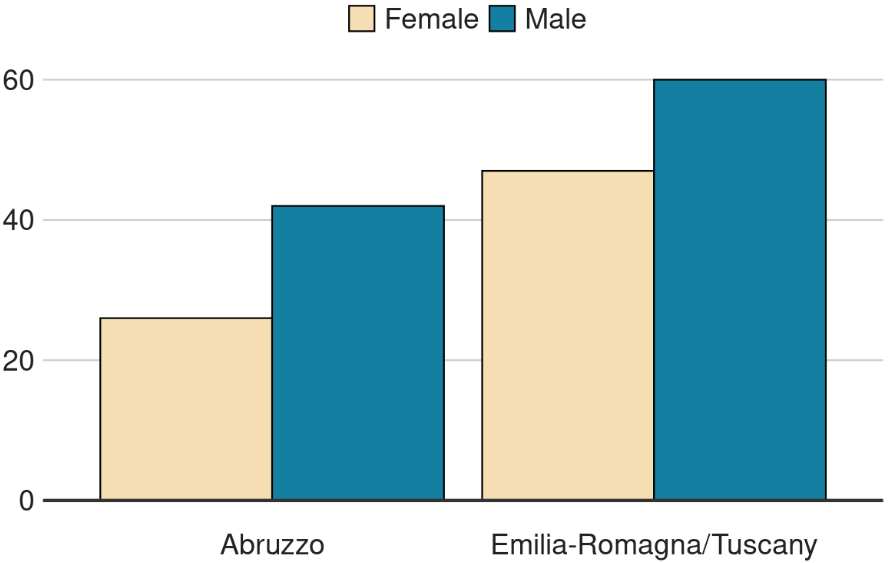

Supplement: S1 Fig — The number of wolves is shown on the y-axis. (PNG) [file pone.0282232.s001.png]

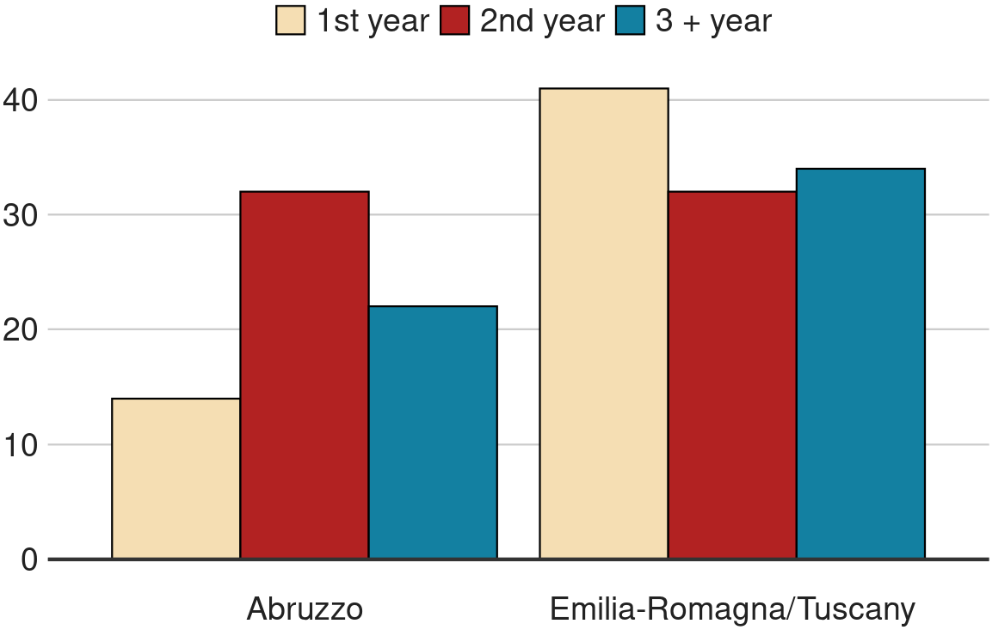

Supplement: S2 Fig — The number of recovered wolves is shown on the y-axis. (PNG) [file pone.0282232.s002.png]

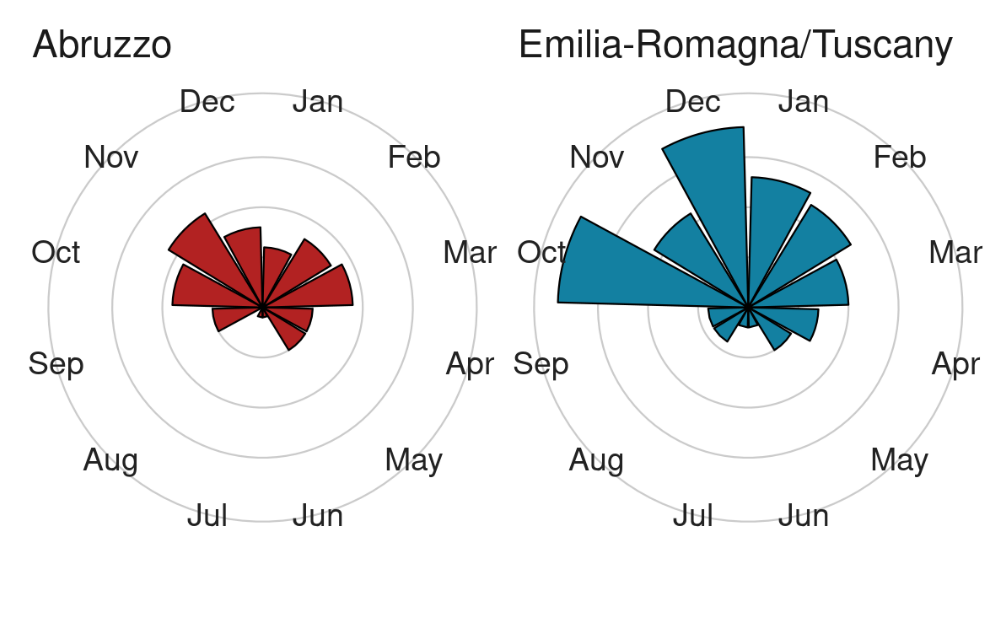

Supplement: S3 Fig — (PNG) [file pone.0282232.s003.png]

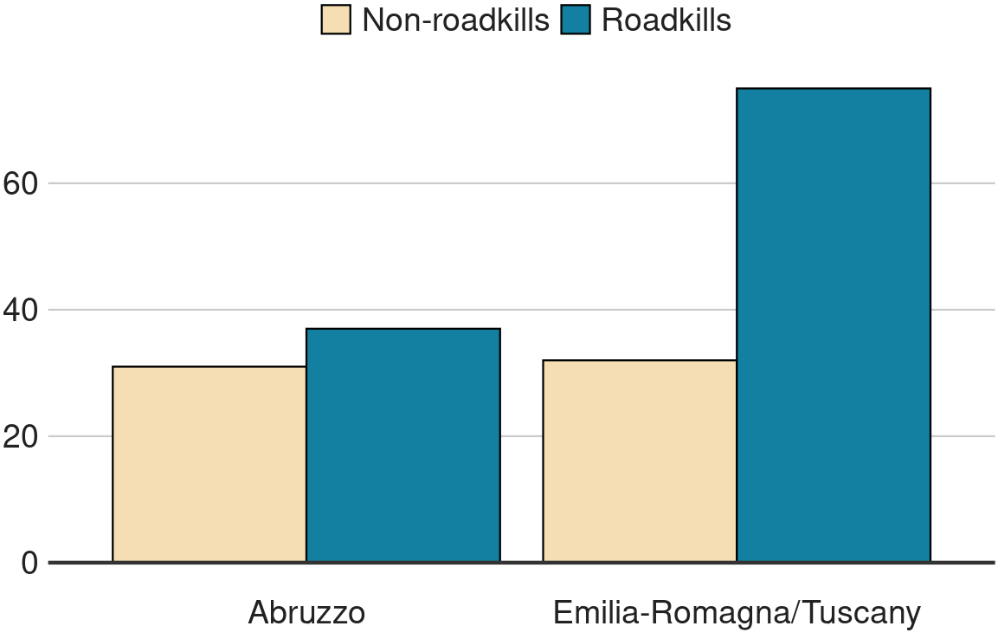

Supplement: S4 Fig — The number of recovered wolves is shown on the y-axis. (PNG) [file pone.0282232.s004.png]

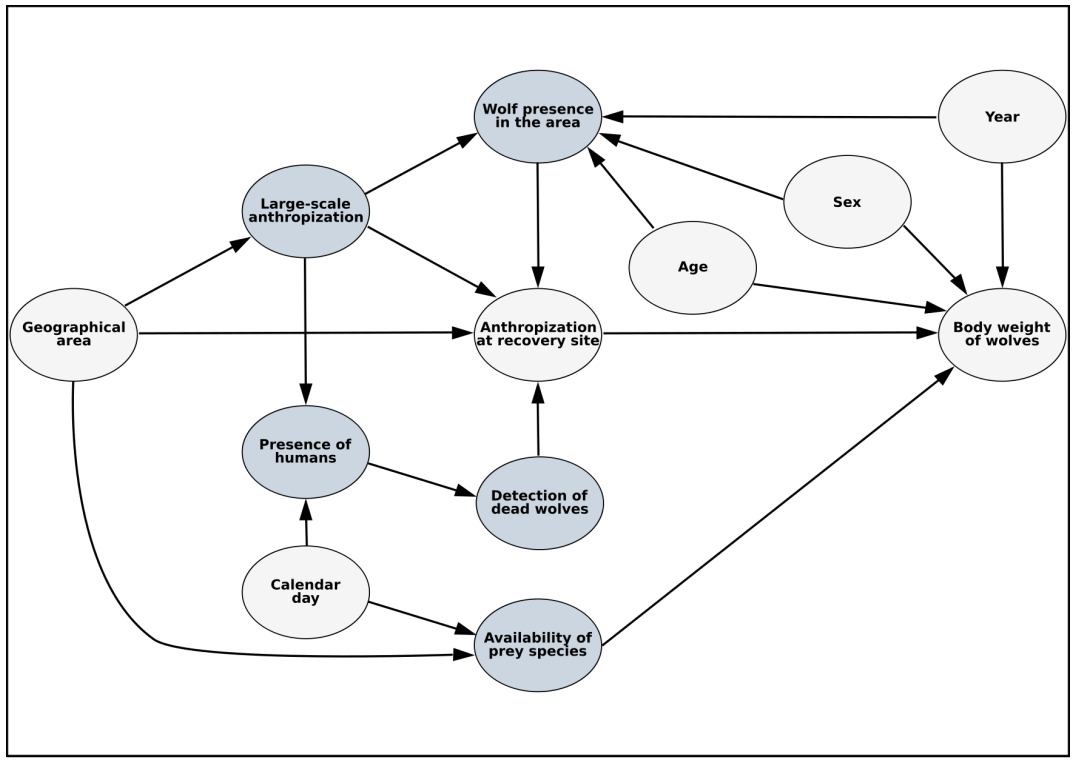

Supplement: S5 Fig — Total body length is not shown, as the predictor was included in the model not as a confounder, but to rule out the part of body mass that did not depend upon body condition, but upon differences in the size of animals. (PNG) [file pone.0282232.s005.png]

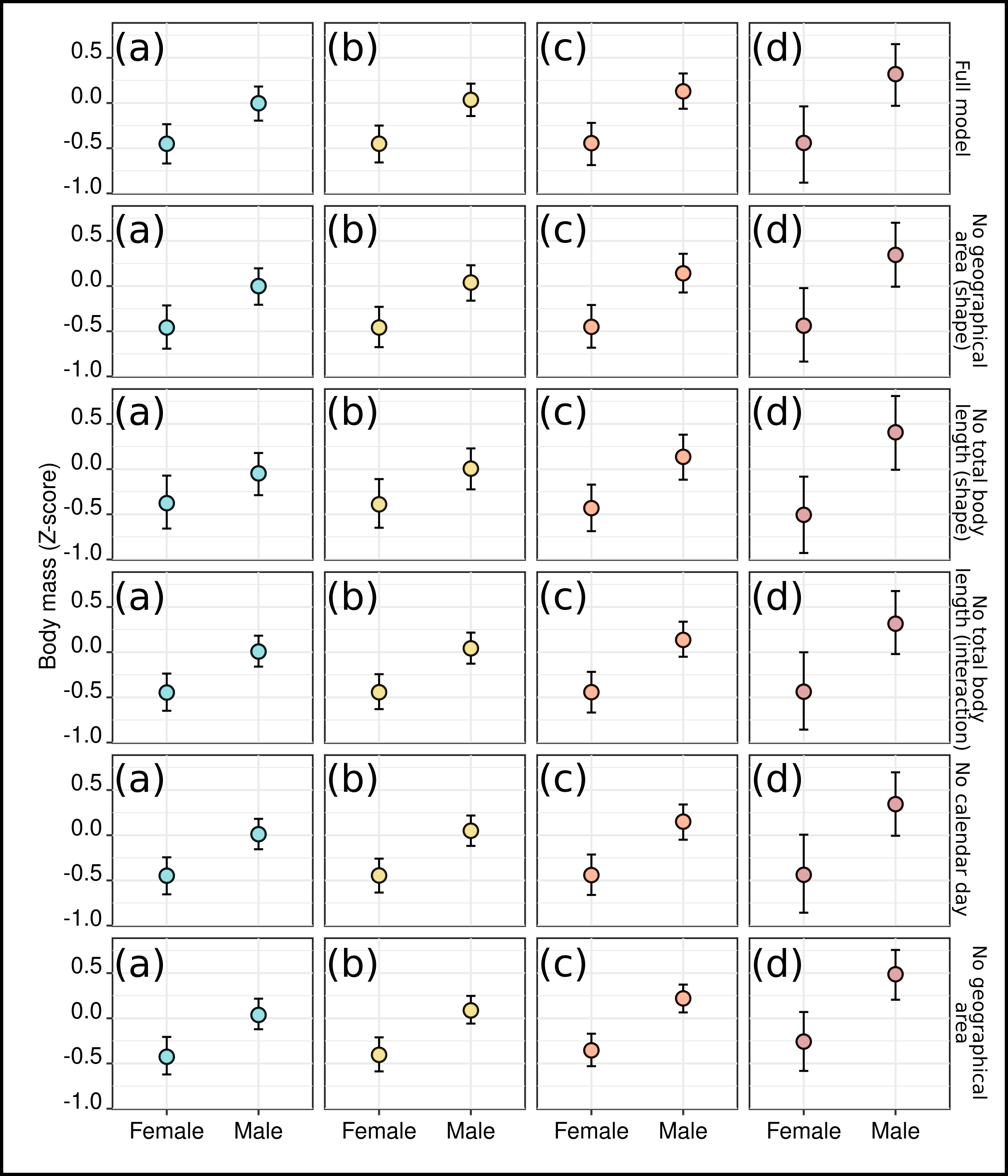

Supplement: S6 Fig — Plots correspond to the first (a), second (b), third (c), and fourth (d) quartiles of the distribution of median Human Footprint Index, calculated in a buffer with a 6-km radius around the point where animals were found. Columns (a) to (d) therefore corresponds to increasingly urbanized areas, while rows to models selected in S1 Table. (PNG) [file pone.0282232.s006.png]

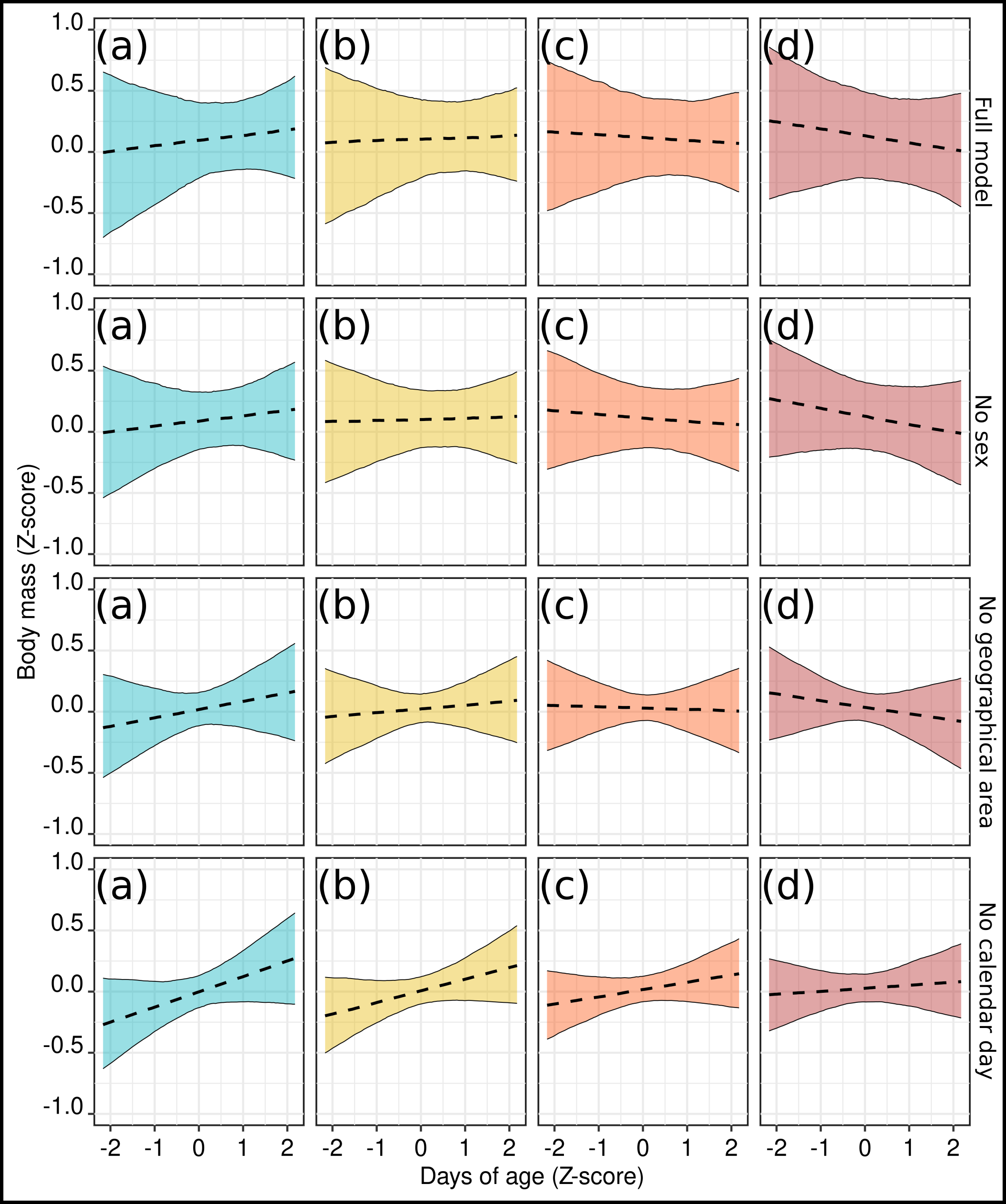

Supplement: S7 Fig — Plots correspond to the first (a), second (b), third (c) and fourth (quartiles) of the distribution of median Human Footprint Index, calculated in a buffer with 6-km radius around the point where animals were found. Columns (a) to (d) therefore corresponds to increasingly urbanized areas, while rows to models selected in S2 Table. (PNG) [file pone.0282232.s007.png]

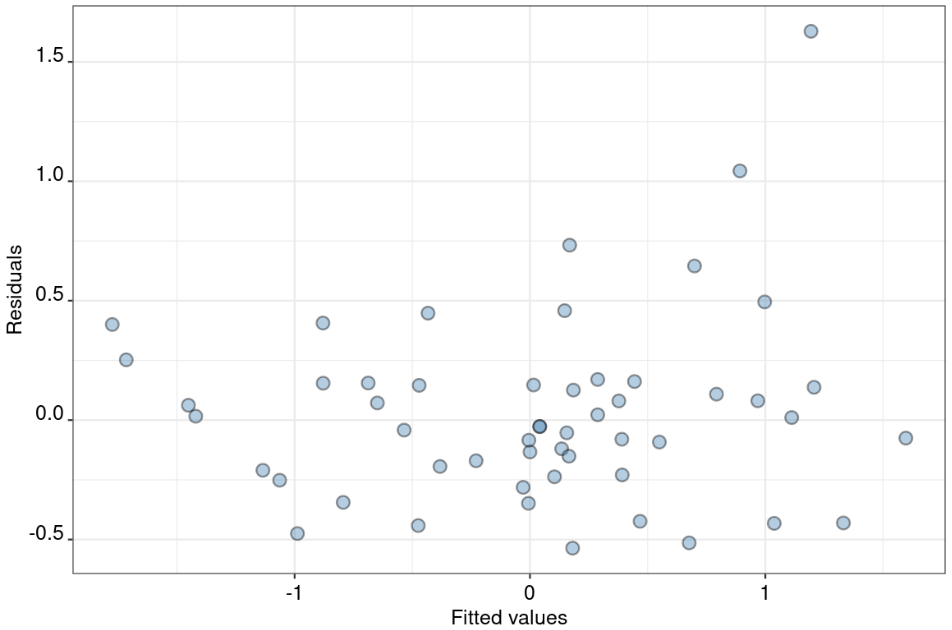

Supplement: S8 Fig — (PNG) [file pone.0282232.s008.png]

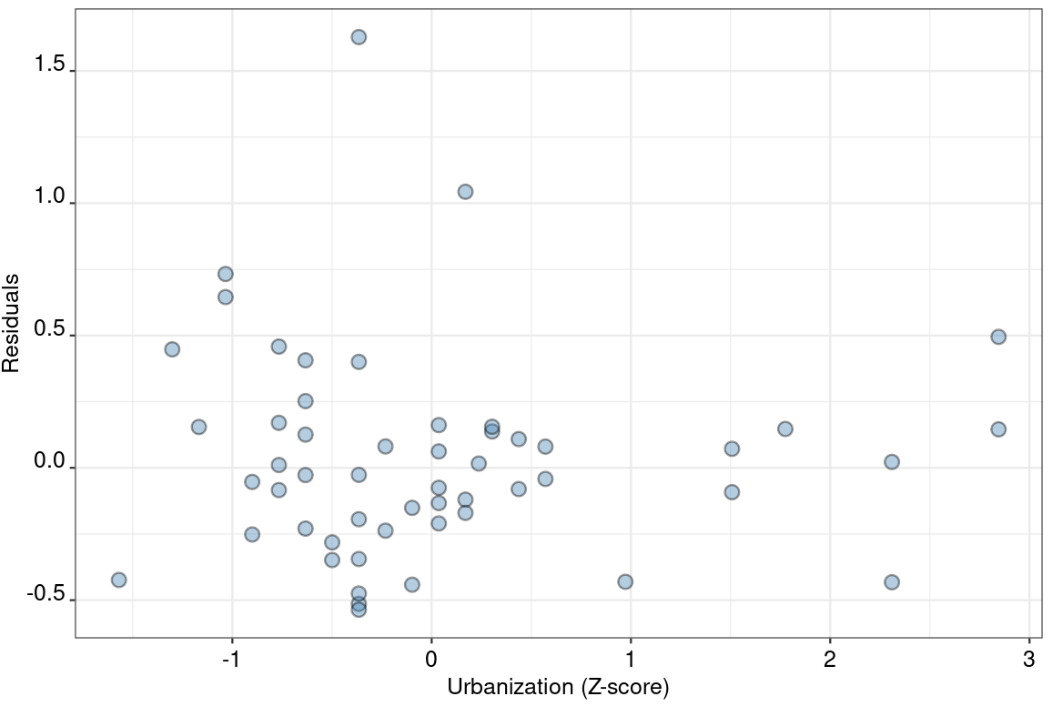

Supplement: S9 Fig — (PNG) [file pone.0282232.s009.png]

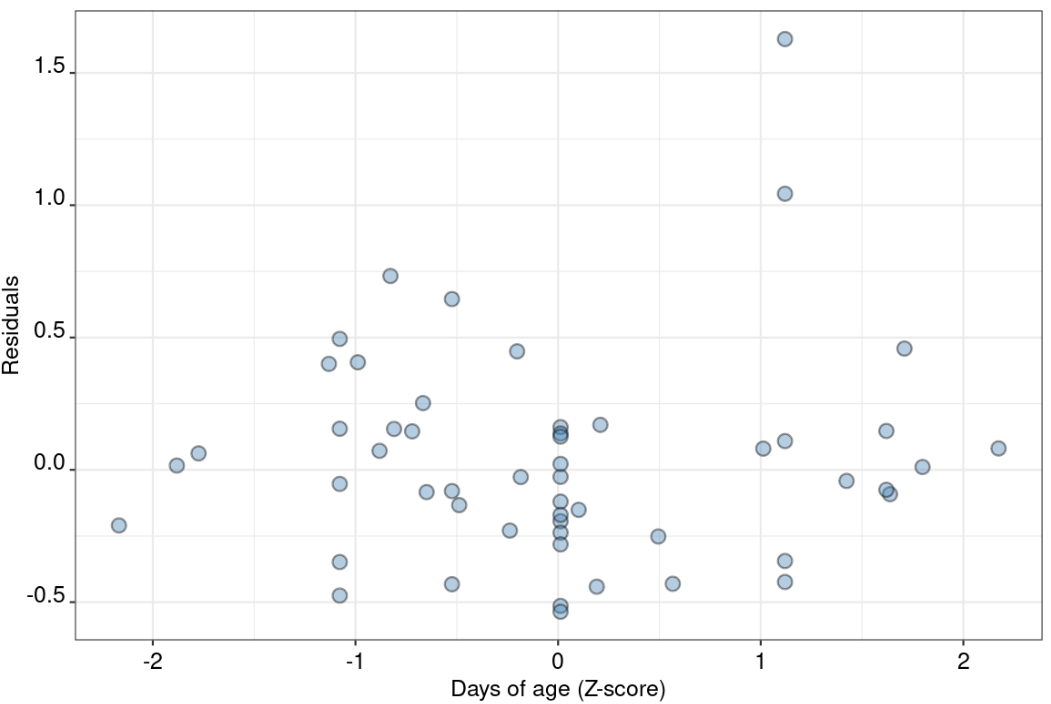

Supplement: S10 Fig — (PNG) [file pone.0282232.s010.png]

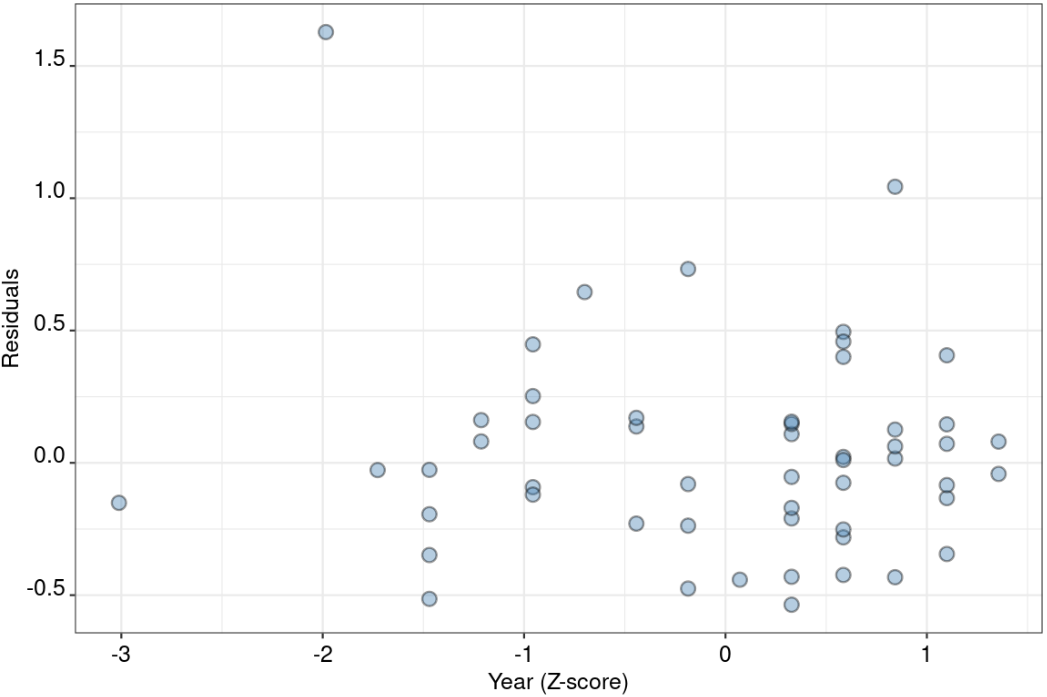

Supplement: S11 Fig — (PNG) [file pone.0282232.s011.png]

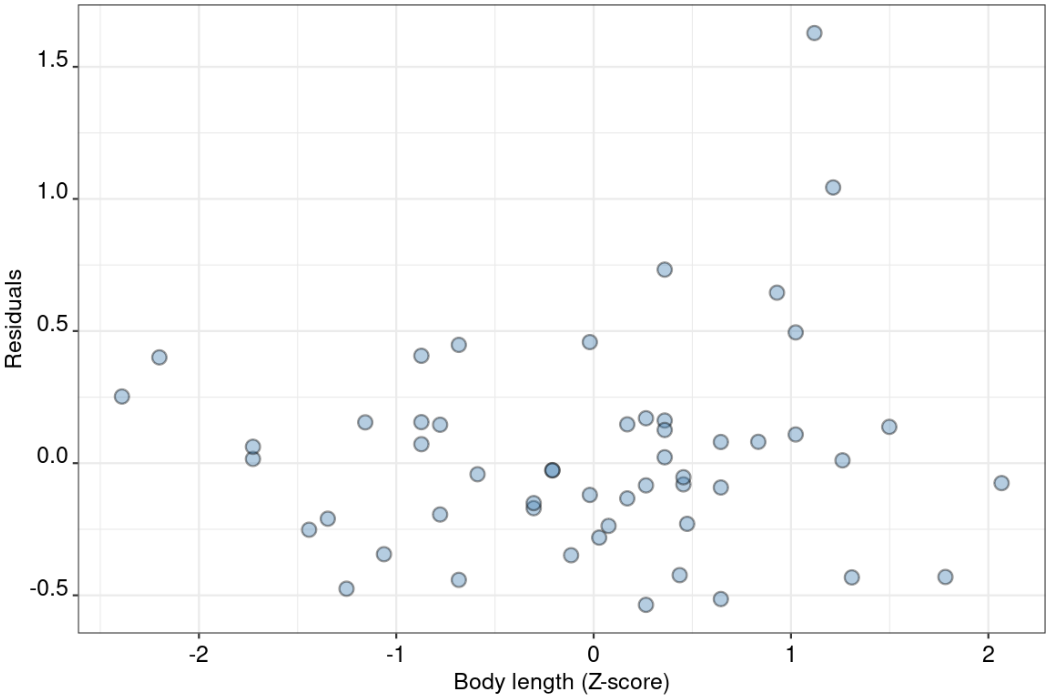

Supplement: S12 Fig — (PNG) [file pone.0282232.s012.png]

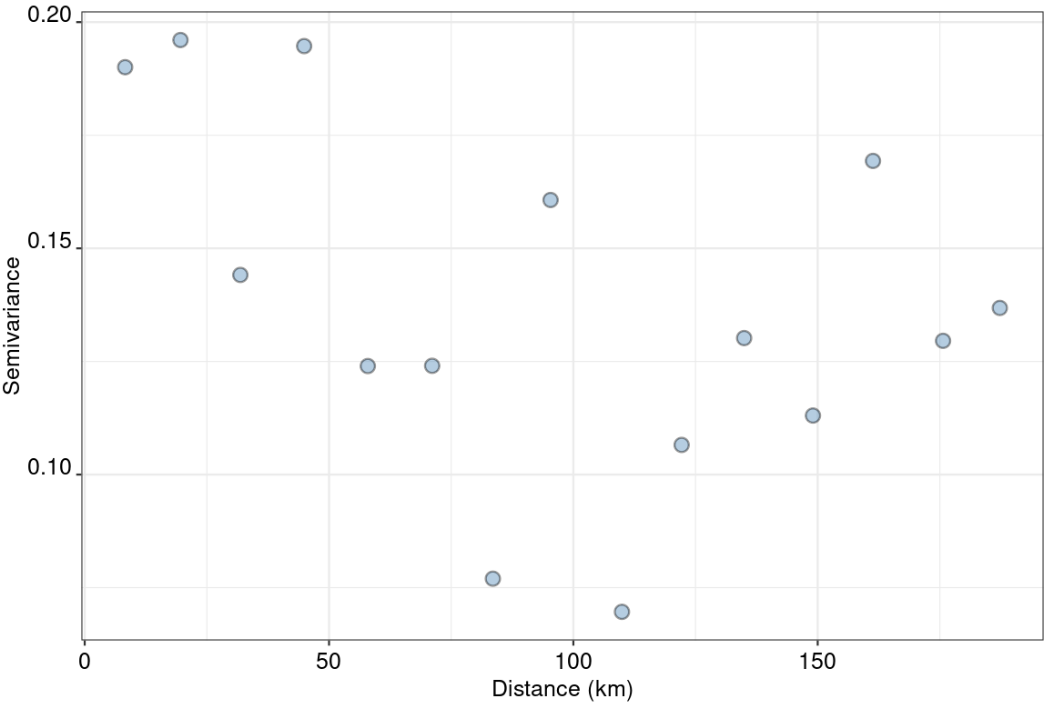

Supplement: S13 Fig — (PNG) [file pone.0282232.s013.png]

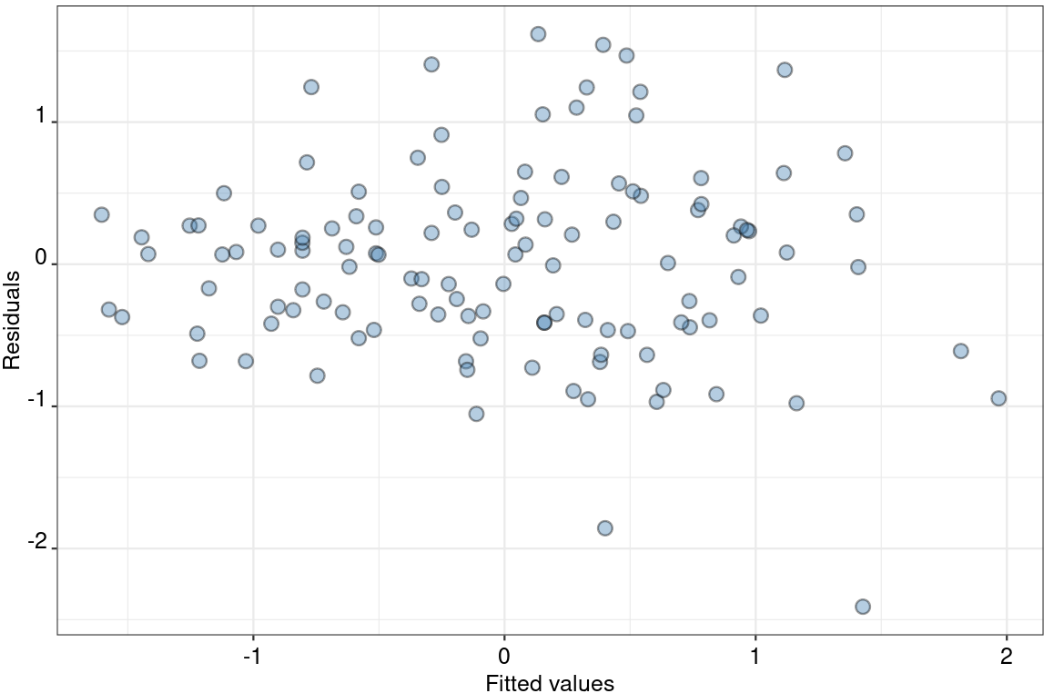

Supplement: S14 Fig — (PNG) [file pone.0282232.s014.png]

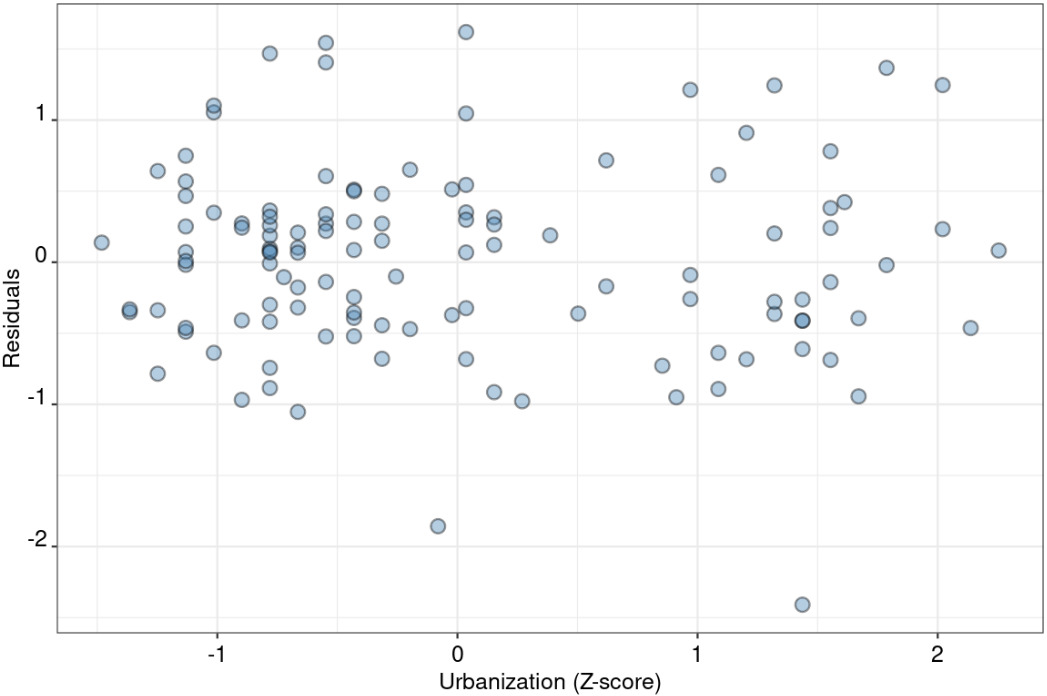

Supplement: S15 Fig — (PNG) [file pone.0282232.s015.png]

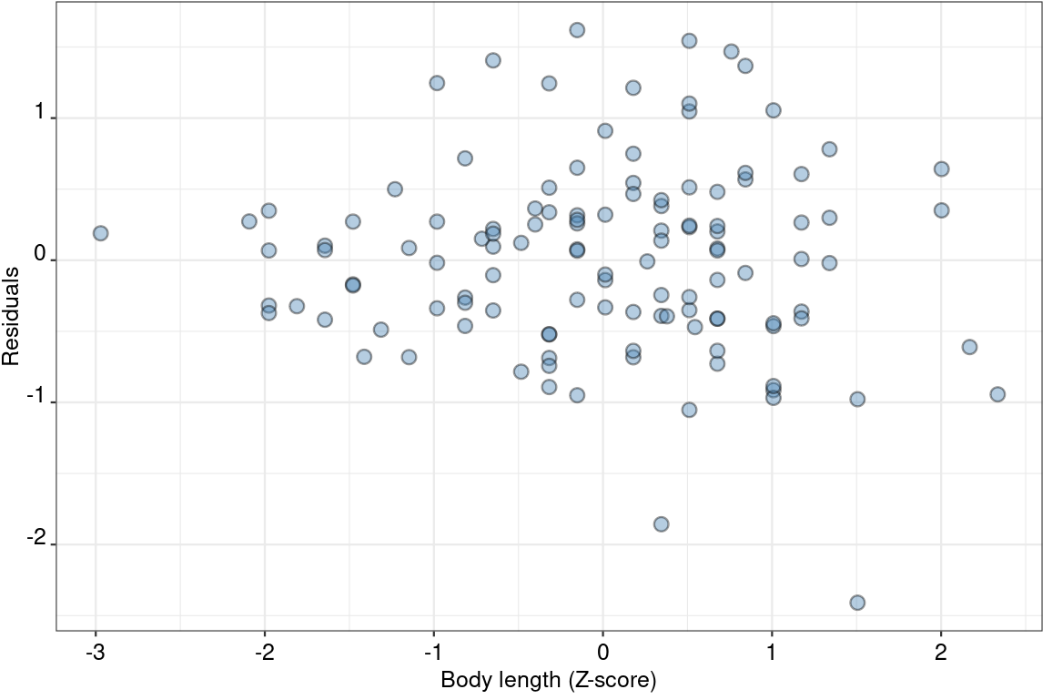

Supplement: S16 Fig — (PNG) [file pone.0282232.s016.png]

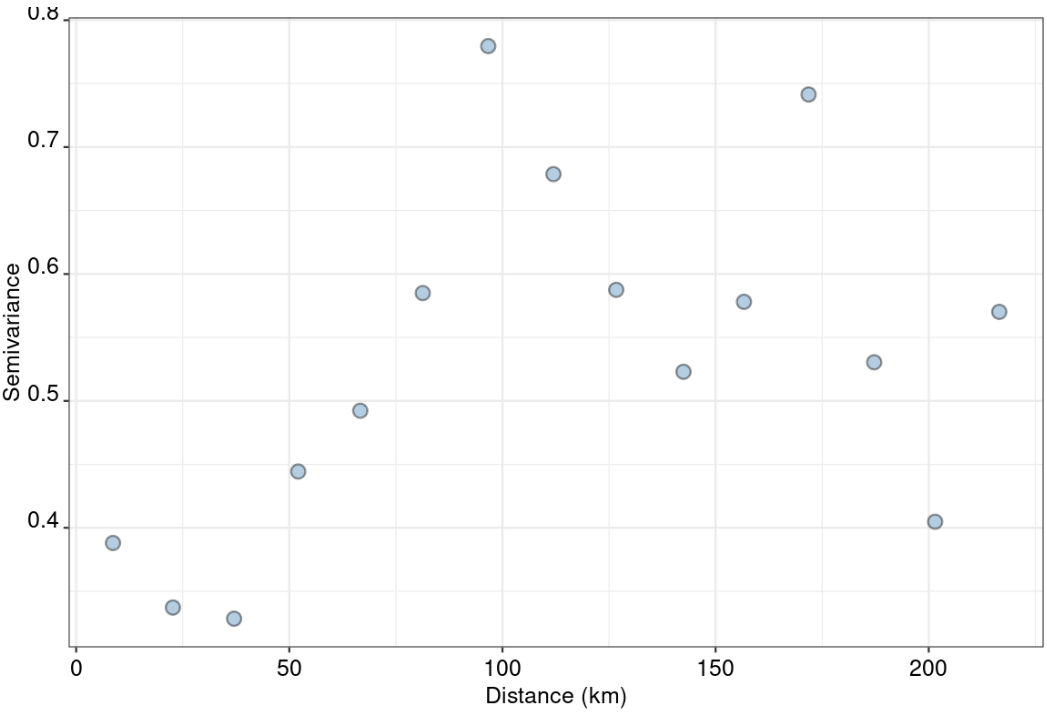

Supplement: S17 Fig — (PNG) [file pone.0282232.s017.png]
