# Supplementary material for: A human-neutral large carnivore? No patterns in the body mass of gray wolves across a gradient of anthropization
Source: PLoS One. 2023 Jun 1;18(6):e0282232. doi: 10.1371/journal.pone.0282232 (PMC10234544; doi:10.1371/journal.pone.0282232)
Supplement: S1 Table — Outputs from models about the body mass of wolves in their first year of age (left). (PDF) [file pone.0282232.s018.pdf]

Model formula	ELPD ± S.E.
Body mass ~ anthropization + age in days + day of the year when animals were found + year when animals were found + area + sex + age in days : anthropization + total body length	-33.1 ± 7.5
Body mass ~ anthropization + age in days + day of the year when animals were found + year when animals were found + area + age in days : anthropization + total body length	-32.0 ± 7.6
Body mass ~ anthropization + age in days + day of the year when animals were found + year when animals were found + age in days : anthropization + total body length	-31.2 ± 7.4
Body mass ~ anthropization + age in days + year when animals were found + age in days : anthropization + total body length	-30.4 ± 8.1
Body mass ~ anthropization + age in days + age in days : anthropization + total body length	-30.5 ± 8.4
