# Supplementary material for: A human-neutral large carnivore? No patterns in the body mass of gray wolves across a gradient of anthropization
Source: PLoS One. 2023 Jun 1;18(6):e0282232. doi: 10.1371/journal.pone.0282232 (PMC10234544; doi:10.1371/journal.pone.0282232)
Supplement: S2 Table — Outputs from models about adult wolves. (PDF) [file pone.0282232.s019.pdf]

Model formula	ELPD ± S.E.
Conditional mean: Body mass ~ anthropization + day of the year when animals were found + year when animals were found + area + sex + sex : anthropization + total body length<br>Conditional variance: total body length + area	-120.9 ± 8.9
Conditional mean: Body mass ~ anthropization + day of the year when animals were found + year when animals were found + area + sex + sex : anthropization + total body length<br>Conditional variance: total body length	-122.9 ± 9.6
Conditional mean: Body mass ~ anthropization + day of the year when animals were found + year when animals were found + area + sex + sex : anthropization + total body length	-126.9 ± 9.4
Conditional mean: Body mass ~ anthropization + day of the year when animals were found + area + sex + sex : anthropization + total body length<br>Conditional variance: total body length + area	-119.9 ± 8.5
Conditional mean: Body mass ~ anthropization + sex + sex : anthropization + total body length<br>Conditional variance: total body length + area	-119.9 ± 8.6
