# Supplementary material for: A human-neutral large carnivore? No patterns in the body mass of gray wolves across a gradient of anthropization
Source: PLoS One. 2023 Jun 1;18(6):e0282232. doi: 10.1371/journal.pone.0282232 (PMC10234544; doi:10.1371/journal.pone.0282232)
Supplement: Attachment — Submitted filename: Response_to_Reviewers_07Feb2023.docx [file pone.0282232.s021.docx]

################################################################################

PONE-D-22-27536R1 - A human-neutral large carnivore? No patterns in the body mass of gray wolves across a gradient of anthropization

################################################################################

Dear Dr. Cerri,

Thank you for submitting your manuscript to PLOS ONE. After careful consideration, we feel that it has merit but does not fully meet PLOS ONE’s publication criteria as it currently stands. Therefore, we invite you to submit a revised version of the manuscript that addresses the points raised during the review process.

Please submit your revised manuscript by Mar 16 2023 11:59PM. If you will need more time than this to complete your revisions, please reply to this message or contact the journal office at plosone@plos.org. When you're ready to submit your revision, log on to https://www.editorialmanager.com/pone/ and select the 'Submissions Needing Revision' folder to locate your manuscript file.

Please include the following items when submitting your revised manuscript:

- A rebuttal letter that responds to each point raised by the academic editor and reviewer(s). You should upload this letter as a separate file labeled 'Response to Reviewers'.
- A marked-up copy of your manuscript that highlights changes made to the original version. You should upload this as a separate file labeled 'Revised Manuscript with Track Changes'.
- An unmarked version of your revised paper without tracked changes. You should upload this as a separate file labeled 'Manuscript'.

If you would like to make changes to your financial disclosure, please include your updated statement in your cover letter. Guidelines for resubmitting your figure files are available below the reviewer comments at the end of this letter.

If applicable, we recommend that you deposit your laboratory protocols in protocols.io to enhance the reproducibility of your results. Protocols.io assigns your protocol its own identifier (DOI) so that it can be cited independently in the future. For instructions see: https://journals.plos.org/plosone/s/submission-guidelines#loc-laboratory-protocols. Additionally, PLOS ONE offers an option for publishing peer-reviewed Lab Protocol articles, which describe protocols hosted on protocols.io. Read more information on sharing protocols at https://plos.org/protocols?utm_medium=editorial-email&utm_source=authorletters&utm_campaign=protocols.

We look forward to receiving your revised manuscript.

Kind regards,

Paulo Corti, Ph.D.

Academic Editor

PLOS ONE

################################################################################

Journal Requirements:

################################################################################

Please review your reference list to ensure that it is complete and correct. If you have cited papers that have been retracted, please include the rationale for doing so in the manuscript text, or remove these references and replace them with relevant current references. Any changes to the reference list should be mentioned in the rebuttal letter that accompanies your revised manuscript. If you need to cite a retracted article, indicate the article’s retracted status in the References list and also include a citation and full reference for the retraction notice.

################################################################################

Additional Editor Comments:

################################################################################

**Comment**: Please answer the questions from reviewer #2. I addition, from the previous review of referee #1, please include the line number of your manuscript in your replay.

**Reply**: we replied in detail to each comment raised by Reviewer #2 and we added line counts, as requested by Reviewer#1.

################################################################################

Reviewers' comments:

################################################################################

1. If the authors have adequately addressed your comments raised in a previous round of review and you feel that this manuscript is now acceptable for publication, you may indicate that here to bypass the “Comments to the Author” section, enter your conflict of interest statement in the “Confidential to Editor” section, and submit your "Accept" recommendation.

Reviewer #2: (No Response)

################################################################################

2. Is the manuscript technically sound, and do the data support the conclusions? - The manuscript must describe a technically sound piece of scientific research with data that supports the conclusions. Experiments must have been conducted rigorously, with appropriate controls, replication, and sample sizes. The conclusions must be drawn appropriately based on the data presented.

Reviewer #2: Yes

################################################################################

3. Has the statistical analysis been performed appropriately and rigorously?

Reviewer #2: Yes

################################################################################

4. Have the authors made all data underlying the findings in their manuscript fully available? - The PLOS Data policy requires authors to make all data underlying the findings described in their manuscript fully available without restriction, with rare exception (please refer to the Data Availability Statement in the manuscript PDF file). The data should be provided as part of the manuscript or its supporting information, or deposited to a public repository. For example, in addition to summary statistics, the data points behind means, medians and variance measures should be available. If there are restrictions on publicly sharing data—e.g. participant privacy or use of data from a third party—those must be specified.

Reviewer #2: Yes

################################################################################

5. Is the manuscript presented in an intelligible fashion and written in standard English? - PLOS ONE does not copyedit accepted manuscripts, so the language in submitted articles must be clear, correct, and unambiguous. Any typographical or grammatical errors should be corrected at revision, so please note any specific errors here.

Reviewer #2: (No Response)

################################################################################

6. Review Comments to the Author - Please use the space provided to explain your answers to the questions above. You may also include additional comments for the author, including concerns about dual publication, research ethics, or publication ethics. (Please upload your review as an attachment if it exceeds 20,000 characters)

**Comment**: Reviewer #2: The corrected manuscript contains additions at several points that I requested in the review. However, I still recommend a few additions. Overall, the reliability of the data is not in question, but the Authors must describe the data quality as precisely as possible, excluding or mentioning influencing and limiting circumstances based on objective criteria. This is important because the Authors' first study on this topic can be a reference for further similar studies.

**Reply**: we addressed each point you raised in detail, and we now hope to have described data quality as precisely as possible. Please, see lines 157-183.

**Comment**: As the Authors write, it is well known that wolf-dog hybrids are present in a considerable proportion of the Italian wolf population. The Reader will also know this; that is, it is not the fact that this question is dealt with in the methodology that causes confusion, but that it weakens the results if it is omitted. In the Methods (Collection of dead wolves and laboratory analysis subchapter), I propose a short addition in which it is described that during the protocol used in the collection of the carcass and the autopsy, based on morphological and other phenotypic characteristics, hybrid-suspicious individuals were excluded. Thus, hybrids did not occur, or in the absence of DNA analysis, they may have been included in the analysis in a small proportion. (If this statement is true.) Please add, even with a new reference, how the taxonomic classification of the dead individuals took place. A higher proportion of hybrids in the examined sample would be problematic.

**Reply**: Our sample did not include a large proportion of hybrids as out of 185 individuals only 11 were not tested genetically and in the other 174 only 8 resulted recent hybrids (F1 or F2), so we have not excluded these individuals from analyses.

Although we acknowledge that this is a limit of our research, and that future studies should explore the extent to which hybridization could affect wolf behavior and fitness in anthropized environments, we did not discard hybrid individuals, because evidence for potential differences is still weak and largely unknown.

While during domestication dogs started producing alpha-amylase (<https://doi.org/10.1038/nature11837>), amylase activity is highly variable between dog breeds and even between individuals of the same breed, due to a strong heterogeneity in the number of copies of the gene AMY2B (<https://doi.org/10.1111/age.12179>). Therefore, provided that for wolves we do not know if AMY2B is associated to amylase activity just like in domestic dogs, hybrid wolves could have simply originated from dogs with a low number of copies of AMY2B, and therefore they could have a low amylase activity and no increased capacity to exploit starch-rich human food waste at all.

As for other behavioral traits, associated to increased boldness towards humans, which dogs developed during domestication, our current understanding of the underlying genetic mechanisms is still limited (e.g., <https://www.frontiersin.org/articles/10.3389/fvets.2021.693290/full>). Also considered that wolves in Italy were also subjected to long-term pressures from prolonged coexistence with humans, a phenomenon which could have masked any effect of hybridization on the selection of “bolder” individuals.

While we acknowledge that these potential mechanisms provide fascinating directions for future studies, and certainly deserve further attention (like we mentioned in the Discussion), we do not deem them to constitute a solid basis of evidence for discarding non-tested wolves from our datasets.

We now reported the number of wolves who were tested for hybridization, as well as the number of recent hybrids in our sample. Moreover, we provided a short description on how we carried out the classification of the dead individuals. Please see lines 168-183.

**Comment**: Lines 269-271 (corrected manuscript). Changes in food sources and the relationship between food sources and body condition were not studied. Any statements related to this topic must be referred to.

**Reply**: we understand that it is important to contextualize this point. We now emphasized, that our analyses dealt with landscape-scales responses and not with fixed or time-variable resource selection. Please see lines 275-277.

**Comment**: Line 277. No other large carnivores than grey wolf were studied; therefore should be mentioned species or rather references here.

**Reply**: we now shortly explained the content of the two references. Please see lines 283-288.

**Comment**: Lines 291-292. Given that no food source analysis was performed, I also recommend citing the literature supporting the statement for this sentence, "This suggests a positive influence of human-derived food sources...".

**Reply**: Unfortunately we found no references that we could cite in support of what we suggested as an hypothesis. There are very few studies exploring the diet of wolf pups in Europe, let alone the diet of wolf pups in anthropized areas. Here we are just hypothesizing a mechanism (we used “this suggests...”) to explain the pattern that we observed, which is plausible considered the that pups depend upon adults in their first months.

**Comment**: Lines 318-320. The relationship between body condition and reproduction was also not included in the analysis of the study, so please cite here the publication based on which this sentence was written.

**Reply**: we now added the reference as requested. See lines 332 and reference n.96.

**Comment**: For the topic of body weight change, further studies on canids could have been considered. (The omnivorous brown bear and mustelids are inappropriate in this respect.) The question has been answered.

**Reply**: We mentioned studies about the effect of anthropogenic food subsidies that dealt with a range of different species, other than wolves, because there is relatively little research on this topic. In the study we tried to focus on potential responses of top predators, what gray wolves are, to anthropization, also by considering the potential exploitation of anthropogenic food sources.

################################################################################

7. PLOS authors have the option to publish the peer review history of their article (what does this mean?). If published, this will include your full peer review and any attached files. - If you choose “no”, your identity will remain anonymous but your review may still be made public. Do you want your identity to be public for this peer review? For information about this choice, including consent withdrawal, please see our Privacy Policy.

Reviewer #2: No
